# Socioeconomic predictors and consequences of depression among primary care attenders with non-communicable diseases in the Western Cape, South Africa: cohort study within a randomised trial

**DOI:** 10.1186/s12889-015-2509-4

**Published:** 2015-11-30

**Authors:** Naomi Folb, Crick Lund, Lara R. Fairall, Venessa Timmerman, Naomi S. Levitt, Krisela Steyn, Max O. Bachmann

**Affiliations:** Knowledge Translation Unit, University of Cape Town Lung Institute, Cape Town, South Africa; Alan J Flisher Centre for Public Mental Health, Department of Psychiatry and Mental Health, University of Cape Town, Cape Town, South Africa; Department of Medicine, University of Cape Town, Cape Town, South Africa; Norwich Medical School, University of East Anglia, Norwich, UK

**Keywords:** South Africa, Primary care, Depression, Social determinants

## Abstract

**Background:**

Socioeconomic predictors and consequences of depression and its treatment were investigated in 4393 adults with specified non-communicable diseases attending 38 public sector primary care clinics in the Eden and Overberg districts of the Western Cape, South Africa.

**Methods:**

Participants were interviewed at baseline in 2011 and 14 months later, as part of a randomised controlled trial of a guideline-based intervention to improve diagnosis and management of chronic diseases. The 10-item Center for Epidemiologic Studies Depression Scale (CESD-10) was used to assess depression symptoms, with higher scores representing more depressed mood.

**Results:**

Higher CESD-10 scores at baseline were independently associated with being less educated (*p* = 0.004) and having lower income (*p* = 0.003). CESD-10 scores at follow-up were higher in participants with less education (*p* = 0.010) or receiving welfare grants (*p* = 0.007) independent of their baseline scores. Participants with CESD-10 scores of ten or more at baseline (56 % of all participants) had 25 % higher odds of being unemployed at follow-up (*p* = 0.016), independently of baseline CESD-10 score and treatment status. Among participants with baseline CESD-10 scores of ten or more, antidepressant medication at baseline was independently more likely in participants who had more education (*p* = 0.002), higher income (*p* < 0.001), or were unemployed (*p* = 0.001). Antidepressant medication at follow up was independently more likely in participants with higher income (*p* = 0.023), and in clinics with better access to pharmacists (*p* = 0.053) and off-site drug delivery (*p* = 0.013).

**Conclusions:**

Socioeconomic disadvantage appears to be both a cause and consequence of depression, and may also be a barrier to treatment. There are opportunities for improving the prevention, diagnosis and treatment of depression in primary care in inequitable middle income countries like South Africa.

**Trial registration:**

The trial is registered with Current Controlled Trials (ISRCTN20283604).

## Background

Depression is a common mental disorder, causing a high level of disease burden. There were an estimated 298 million cases of major depressive disorder worldwide in 2010 [1] and this disorder was ranked the second leading cause of years lived with disability (YLD) [1, 2].

Mental disorders are also an important cause of disease burden in South Africa. The South African Stress and Health (SASH) study indicated a lifetime prevalence of major depression of 9.7 % and a 12 month prevalence of 4.9 % [3].

Approximately 80 % of South Africans are estimated to be dependent on public health sector services [4], which are inadequately equipped to address the high prevalence of mental disorders. There is marked under-treatment of mental disorders in primary care in South Africa. Three quarters of adults with a mental disorder in the SASH study received no treatment in the year of the interview [5]. This treatment gap is consistent with evidence from many low and middle income countries (LMICs) [6].

South Africa is one of the most unequal countries in the world [7], with wide disparities in wealth and health [8]. Income inequality has been shown to be positively associated with mental illness [9–11]. A study among older Americans found those living in counties with higher income inequality were more depressed, independent of their demographic characteristics, socioeconomic status, and physical health [10]. In South Africa, the burden of ill-health has been demonstrated to be greater among lower socio-economic groups [12].

A number of studies in LMICs have shown an association between indicators of poverty and mental disorders [11, 13]. A systematic review of the relationship between poverty and common mental disorders in LMICs found a relatively consistent and strong association between common mental disorders and education, food insecurity, housing, social class, socio-economic status and financial stress; whereas income, employment and consumption were found to be more equivocal [11]. A second systematic review of poverty and common mental disorders in developing countries found most studies showed an association between risk of common mental disorders and low levels of education, and many studies also showed a relationship with other indicators of poverty such as poor housing or low income [13].

Associations have been found between depression and non-communicable diseases. High depression scores have been found to be an independent risk factor for hypertension, and there is evidence for an association between mental disorder and diabetes. In addition, depression has been shown to be associated with poor glycaemic control [14].

Depression has been shown to be associated with more frequent exacerbations in patients with chronic obstructive pulmonary disease (COPD), worse short-term survival, and higher rates of post-exacerbation readmission to hospital. An interaction effect has also been reported between symptoms of depression and death among patients with COPD [15].

The majority of past studies looking at socio-economic associations with depression in LMICs have been cross-sectional, making it difficult to draw conclusions on causality [11]. Two explanations have been proposed for the inverse relationship between psychiatric disorders and socioeconomic status. Social causation postulates that adversity and stress due to conditions of poverty increase the risk of mental illness, whereas social selection/drift postulates that people with mental illness are at increased risk of drifting into or remaining in poverty due to factors such as loss of employment, reduced productivity, stigma and increased health expenditure [16]. Although these causal pathways are complex, evidence suggests social causation may be more important for common mental disorders such as depression, particularly in women, while social selection/drift processes may be more important for schizophrenia [17].

Few studies have addressed predictors of change in depression symptoms, and predictors of change in treatment for depression over time in LMICs. In addition, few studies have explored the health and economic impact of depression over time.

The aims of this cohort study were to investigate the extent to which socioeconomic position and physical illness (hypertension, diabetes, chronic respiratory disease) predict depression symptoms over time among primary care attenders, and the extent to which these factors and health service characteristics predict treatment of depression over time.

## Methods

### Study design and context

This paper reports on a cohort study within a cluster randomised controlled trial (RCT), including cross-sectional baseline data and longitudinal data on changes from baseline to follow up. The aim of the RCT was to evaluate the effectiveness of the Primary Care 101 guideline training programme for primary health care providers [18, 19], and to assess whether the programme improved quality of care for specified chronic diseases. Primary Care 101 consists of three elements: a 101-page algorithmic guideline that covers common symptoms and conditions in adults; an educational outreach programme in which nurse trainers deliver interactive training sessions on-site to all staff at a facility, using the Primary Care 101 guideline and case scenarios; and additional prescribing provisions for nurses who successfully complete their training.

Thirty-eight clinics in the Eden and Overberg districts of the Western Cape, South Africa, were cluster randomised either to receive the Primary Care 101 training programme for health care providers, or to continue with usual care. Eligible patients, defined below, who provided consent were interviewed at baseline in 2011 and once more, 14 months later [19]. The analyses for this study included data from the whole RCT cohort at baseline and follow-up, combining the intervention and control arms.

### Study population and sample

The study population comprised adults attending public sector primary care clinics in two districts of the Western Cape province of South Africa. The communities served by the public sector clinics in these two districts are characterised by high levels of unemployment and socio-economic deprivation. In 2011, unemployment rates were 22.5 and 17.0 % in the Eden and Overberg districts respectively [20], and the Eden district was rated as the poorest in the Western Cape province [21]. The study site is typical of many low resource rural and small urban settings in South Africa, in which the public sector primary health care clinics are nurse-led with some doctor support.

Thirty-eight of the largest primary care clinics in the Eden district and two Overberg sub-districts were selected. Each clinic services at least 10 000 attendances per year and they are staffed by nurse practitioners, doctors and community health workers. The study population was restricted to adults 18 years or older, planning to reside in the area for the next year, and capable of actively engaging in an interviewer-administered questionnaire at the time of recruitment.

Among patients who met these criteria, four groups representing patients with hypertension, diabetes, chronic respiratory disease and depression were identified. Patients were eligible for the hypertension and diabetes groups if they reported being on medication for hypertension or diabetes respectively. They were eligible for the respiratory group if they reported being on medication for chronic respiratory disease, or had symptoms of chronic respiratory disease and were not on treatment for tuberculosis. Patients were eligible for the depression group if they scored ten or more on the 10-item Centre for Epidemiologic Studies Depression Scale (CESD-10) [22]. Patients may have fulfilled inclusion criteria for more than one disease group. Participants were sampled consecutively within each clinic and invited to participate in the study, until the sample size required for each clinic was obtained. They were screened for eligibility by orally questioning them and, if they met the eligibility criteria, were then asked to provide informed consent to participate.

### Data collection and coding

At baseline trained fieldworkers administered the electronic questionnaire and took clinical measurements after eligible participants provided informed consent. The baseline questionnaire included questions about demographic characteristics, comorbidities, and socio-economic factors. Participants were asked about the highest level of education they had achieved (no schooling, grade 1–7, grade 8–12 or tertiary/diploma), their employment status (employed, self-employed, student/learner or unemployed), and their employed and pension/grant income in the last month.

The presence of depression symptoms was assessed with the 10-item CES-D scale which was administered to all participants. The 20-item CES-D was originally developed by Radloff (1977) to measure symptoms of depression in the general population [23, 24]. A shortened 10-item version was created by Andresen et al. [22] The CESD-10 items are: “1. I was bothered by things that usually don't bother me. 2. I had trouble keeping my mind on what I was doing. 3. I felt depressed. 4. I felt that everything I did was an effort. 5. I felt hopeful about the future. 6. I felt fearful. 7. My sleep was restless. 8. I was happy. 9. I felt lonely. 10. I could not get going.” The individual items are scored from 0 (rarely or none of the time) to 3 (most of the time) and a score is assigned by totalling all item scores. The possible range of scores is 0–30 for the 10-item scale, with higher scores representing greater degrees of depressed mood [22]. Both the 10- and 20- item CES-D have been used and validated in a number of countries including among HIV infected individuals in South Africa [25, 26].

All participants were asked if they had received psychological counselling in the year leading up to their baseline interview. Counselling was defined as talking with someone in a way that helps to find solutions to problems, or receive emotional support, and not just receiving advice on how to take medication. Participants who reported receiving counselling from a mental health nurse, clinic counsellor, social worker, psychiatrist or psychologist were considered to have received counselling. Participants who reported receiving counselling from a mental health nurse, psychiatrist or psychologist were considered to have been referred to psychiatric services.

Chronic medication prescribed at the time of each participant’s interview for depression, hypertension, diabetes and respiratory disease was recorded. Fieldworkers photocopied all available prescription charts for the year preceding the interview. The trial manager (NF) analysed the prescription charts to identify medication for chronic conditions prescribed for each participant at the time of their interview.

It is common practice in the Eden and Overberg districts for amitriptyline or imipramine to be prescribed at a low dose (25 mg daily) for pain management and insomnia. We considered amitriptyline and imipramine at a dose less than 50 mg daily to be sub-therapeutic for depression [6]. Other antidepressants were not prescribed at sub-therapeutic doses [27]. We therefore defined being on an antidepressant at a therapeutic dose as prescription of amitriptyline or imipramine of 50 mg or more daily, or on any other antidepressant.

Disease-specific control indicators were measured at baseline and follow-up [19]. Systolic and diastolic blood pressure were measured in all participants. Ten year risk of cardiovascular deaths was calculated, based on age, sex, systolic blood pressure, smoking status, reported diabetes and body mass index [28]. The severity of respiratory disease was assessed with the Symptom and Activity domains of the St Georges Respiratory Questionnaire (SGRQ) [29] in participants enrolled in the respiratory disease group. Glycated haemoglobin (HbA1c) was measured in a sub-sample of 704 diabetic participants from 20 randomly selected clinics.

The following clinic characteristics were identified at baseline: availability of a pharmacist, availability of drug supply away from clinic, psychiatric nurse at clinic, doctor at clinic every day, clinic location, clinic patients per year, clinic patients per nurse per year, and intervention versus control clinic.

At follow-up the questionnaire, clinical measurements and prescription data were collected and recorded as for the baseline data. Baseline data collection began in March 2011 and ended in October 2011. Follow-up data collection started in May 2012 and ended in January 2013.

### Statistical methods

The statistical analyses investigated associations between participants’ health and socioeconomic indicators, and their symptoms and treatment of depression. We also investigated associations between depression symptoms reported at baseline and subsequent changes in participants’ income and employment, ten year risk of death from cardiovascular disease and, in participants with hypertension, diabetes, or respiratory disease, in blood pressure control, glycaemic control and respiratory symptoms respectively. Analyses of treatments included the following clinic characteristics as potential explanatory variables: pharmacist in clinic, drug supply available away from clinic, psychiatric nurse at clinic, doctor at clinic every day, clinic location, clinic patients per year, clinic patients per nurse per year, and intervention versus control clinic. These clinic characteristics were investigated because they could potentially influence access to necessary treatment directly, or be indirect indicators of the quality of care.

In all analyses the study’s cluster sampling design was accounted for in regression models with robust adjustment for intra-clinic cluster correlation of outcomes, using Stata version 12.0 statistical software [30]. A *p* value 0.05 or less was considered statistically significant.

Intervention or control arm of the randomised controlled trial was accounted for in all longitudinal analyses. Variables independently associated with the outcome in each model were selected using backwards stepwise selection. At each step, explanatory variables with a p value of less than 0.10 were removed from each model. The purpose of stepwise selection of explanatory variables for each model was to estimate the effects of each socioeconomic indicator without confounding by other socioeconomic indicators or patient characteristics. Even though all of the socioeconomic indicators could theoretically have causally influenced depression and its care, it was not appropriate to keep all of them in every model because of the likelihood that overadjustment for collinear variables would obscure relevant associations.

The primary analyses of variables associated with depression symptoms were multiple linear regression models with CESD-10 score as the continuous outcome variable. Secondary analyses of depression symptoms used multiple logistic regression models with CESD-10 scores coded as high (greater than or equal to ten) or low (less than ten).

Analyses with treatments (antidepressant medication, counselling, or referral to psychiatric services) as outcomes were confined to participants with CESD-10 scores greater than or equal to ten at baseline and used multiple logistic regression models. Primary analyses of antidepressant medication coded treatment as present only if drug doses were defined as therapeutic. Secondary analyses coded antidepressant treatment as present at any dose.

Longitudinal data analysis was as follows. Changes between baseline and follow-up in depression symptoms, antidepressant medication, employment or income used analysis of covariance (ANCOVA) in the multiple regression models, that is, with the follow-up variable as outcome and with the baseline variable as a covariate. This was done to account for regression to the mean, that is, individuals with exceptionally high or low values at baseline would at follow-up tend to have values closer to the mean, due to chance alone [31]. Analyses of changes all included trial arm as a potential explanatory variable.

The trial is registered with Current Controlled Trials (ISRCTN20283604). Ethical approval for the trial was obtained from the University of Cape Town Human Research Ethics Committee and the Western Cape Provincial Department of Health. All participants provided informed consent to participate in the study.

## Results

A total of 4393 participants were enrolled at baseline, of whom 90.5 % were followed up. Prescription records were available for 4364 (99.3 %) participants at baseline and 4284 (97.5 %) participants at follow up.

Table 1 shows the socio-demographic characteristics of participants at baseline. The majority of participants (73 %) were women and half were over the age of 50 years. Seventy-four percent had hypertension, 42 % had diabetes, 26 % had chronic respiratory disease or symptoms and 56 % had CESD-10 scores of ten or more. The majority of participants had not completed secondary school education (52 %), were unemployed (75 %) and receiving a welfare grant (58 %). The average monthly income was equivalent to about US$4.90 per day in 2011 [32], but this includes 26 % who reported having no income. These socioeconomic indicators were all significantly associated with each other, except that non-grant income was not associated with language group.

**Table 1:** Participants’ baseline characteristics and associations with baseline CESD-10^a^ scores

	Number^b^	Percent^b^	CESD-10 Mean	CESD-10 SD	*p* ^c^
Health indicators					
Age (years): mean (SD)	51.6 (13.5) *n* = 4393				<0.001
Sex					<0.001
•Women	3193	72.7	11.2	6.4	
•Men	1199	27.3	9.7	6.2	
Hypertension					<0.001
•No	1166	26.6	12.8	6.2	
•Yes	3226	73.5	10.0	6.3	
Diabetes					<0.001
•No	2551	58.1	11.8	6.4	
•Yes	1841	41.9	9.3	6.0	
Chronic respiratory Disease					<0.001
•No	3235	73.7	10.2	6.3	
•Yes	1157	26.3	12.2	6.4	
CESD-10 score ≥10					
•<10	1926	43.9	4.9	2.8	
•≥10	2466	56.2	15.3	4.3	
Antidepressant, any dose					<0.001
•No	3545	81.3	10.2	6.1	
•Yes	818	18.8	13.2	7.0	
Antidepressant, therapeutic dose					<0.001
•No	3971	91.0	10.3	6.1	
•Yes	392	9.0	15.1	7.3	
Socioeconomic indicators					
Language					0.88
•Afrikaans	3679	83.8	10.8	6.6	
•Xhosa	337	7.7	10.1	5.3	
•English	376	8.6	10.6	5.3	
Highest education					0.35
•None	291	7.3	10.8	6.2	
•Primary	1757	44.2	11.0	6.2	
•Secondary	1853	46.6	10.6	6.5	
•Tertiary	75	1.9	9.9	6.4	
Total monthly income (Rand): mean (SD)	1084 (1254) *n* = 4378				<0.001
Unemployed					0.12
•No	1096	25.0	10.4	6.4	
•Yes	3282	75.0	10.9	6.4	
Welfare grant					0.87
•No	1850	42.3	10.7	6.4	
•Yes	2528	57.7	10.8	6.4	
Housing density (occupants/rooms): mean (SD)	1.8 (1.2) *n* = 2930				<0.001

^a^ *CESD-10* 10-item Center for Epidemiologic Studies Depression Scale

^b^ Except mean and standard deviation (SD) for continuous variables

^c^ Linear regression models adjusted for cluster sample design

Baseline CESD-10 scores had a mean value of 10.8 units (standard deviation (SD) 6.4, median 11, interquartile range 6 to 15). Change in CESD-10 scores had a mean value of 3.1 units (SD 7.0, median 7, interquartile range −1 to 8). Both baseline and change in CESD-10 score had symmetrical bell-shaped distributions, except that the baseline score was truncated at zero. However both distributions were significantly different from Normal according to Stata’s combined skewness and kurtosis tests for Normality. At baseline, CESD-10 scores were positively associated with female sex, chronic respiratory disease, antidepressant use and housing density, and were inversely associated with age, hypertension, diabetes and income.

Linear regression models estimated the independent associations of CESD-10 scores with the health and socioeconomic indicators (Table 2). Baseline CESD-10 scores were higher in participants who had chronic respiratory disease, were unemployed or receiving a welfare grant, and were lower in participants who were older, male, had hypertension or diabetes, were more educated or had higher incomes at baseline. CESD-10 scores at follow-up had increased since baseline in participants who had chronic respiratory disease, spoke Xhosa, or received welfare grants. CESD-10 scores at follow-up were lower in participants who were older, male, had hypertension, or were more educated at baseline.

**Table 2:** Patients’ baseline characteristics independently associated with CESD-10^a^ score at baseline and with change^b^ in CESD-10 score: linear regression models

Outcome	Baseline CESD-10^a^ score				Follow-up CESD-10^a^ score^b^			
Explanatory variable	Coefficient	95 % CI^a^		*p*	Coefficient	95 % CI^a^		*p*
Age (per year)	−0.06	−0.08	−0.04	<0.001	−0.06	−0.08	−0.04	<0.001
Men vs. women	−1.66	−2.19	−1.13	<0.001	−0.96	−1.46	−0.46	<0.001
Hypertension	−1.93	−2.60	−1.27	<0.001	−0.53	−1.07	0.00	0.052
Diabetes	−1.75	−2.27	−1.24	<0.001				
Chronic respiratory disease	1.21	0.51	1.91	<0.001	1.06	0.58	1.54	<0.001
Highest education				0.004^c^				0.010^c^
•None (reference)	1.00				1.00			
•Primary	−0.24	−1.31	0.84	0.656	−0.35	−1.34	0.63	0.473
•Secondary	−1.19	−2.33	−0.05	0.042	−1.48	−2.50	−0.47	0.005
•Tertiary	−0.93	−2.70	0.83	0.291	−1.64	−3.03	−0.25	0.022
Language								0.038^c^
•Afrikaans (reference)					1.00			
•Xhosa					1.90	0.42	3.37	0.013
•English					1.66	−0.66	3.98	0.156
Income (per 1000 Rand per month)	−0.23	−0.37	−0.08	0.003				
Unemployed	0.53	−0.08	1.15	0.086				
Welfare grant baseline	0.54	−0.02	1.10	0.060	0.66	0.19	1.13	0.007
Baseline CESD-10 score^b^	NA				0.32	0.27	0.37	<0.001

^a^ *CESD-10* 10-item Center for Epidemiologic Studies Depression Scale, *CI* confidence interval, *NA* not applicable

^b^ Change modelled with analysis of covariance, that is, with baseline value as covariate

^c^ Wald test for all categories of variable

The secondary analyses mostly confirmed the robustness of the results reported in Table 2, as follows. An equivalent logistic regression model with higher (greater than or equal to ten) versus lower baseline CESD-10 scores as binary outcome found the same variables as in Table 2 to be significant predictors, except that language was not significant (*p* = 0.360), and employment was (*p* = 0.013). Housing density was not independently associated with CESD-10 score as a continuous outcome variable (*p* = 0.148), but was associated with higher CESD-10 score modelled as a binary outcome variable (*p* = 0.05). Logistic regression with higher CESD-10 score at follow-up as a binary outcome variable, adjusted for baseline CESD-10 score, found age, sex, chronic respiratory disease, education and welfare grant, but not hypertension, to be significant predictors. Greater housing density was independently associated with increasing CESD-10 scores in linear and in logistic regression models.

Participants with CESD-10 scores of ten or more at baseline had 25 % higher odds of being unemployed at follow-up, and had R55 higher income per month from welfare grants at follow-up, independently of their employment status or grant income at baseline, and other confounding variables (Table 3). Baseline CESD-10 scores were not independently associated with changes in non-grant income or total income.

**Table 3:** Patient characteristics independently associated with changes^a^ in unemployment and welfare grant income: logistic and linear regression models

Outcome	Unemployed at follow-up^b^				Monthly welfare grant income at follow up (Rand)^c^			
Explanatory variable	OR^d^	95 % CI		*p*	Coefficient	95 % CI^d^		*p*
CESD-10 score ≥10 at baseline	1.25	1.04	1.51	0.016	55	18	91	0.004
Age (per year)	1.05	1.04	1.06	<0.001	9	7	11	<0.001
Men vs. women	0.70	0.57	0.86	0.001				
Chronic respiratory disease					66	25	108	0.003
Diabetes					27	0	55	0.048
Highest education				<0.001^e^				0.017 ^e^
•None (reference)	1.00				0			
•Primary	0.78	0.54	1.13	0.186	−38	−85	8	0.105
•Secondary	0.59	0.38	0.90	0.014	−67	−115	−19	0.007
•Tertiary	0.19	0.10	0.35	<0.001	3	−212	218	0.975
Language				0.052^e^				<0.001^e^
•Afrikaans (reference)	1.00				0			
•Xhosa	0.68	0.46	1.00	0.047	−158	−228	−88	<0.001
•English	1.21	0.85	1.73	0.288	−99	−162	−37	0.003
Unemployed at baseline^a^	13.9	10.7	18.2	<0.001				
Grant income at baseline (per 1000 Rand per month)^a^					602	522	682	<0.001

^a^ Change modelled with analysis of covariance, that is, with baseline value as covariate

^b^ Logistic regression model

^c^ Linear regression model

^d^ *OR* odds ratio, *CI* confidence interval

^e^ Wald test for all categories of variable

Baseline CESD-10 score, whether coded as a continuous or binary variable, was not associated with changes in blood pressure control, glycaemic control, respiratory symptom score or ten year risk of death from cardiovascular disease.

Logistic regression models estimated the independent effects of participant and clinic characteristics on antidepressant medication at baseline and follow-up, among participants with baseline CESD-10 scores of ten or more, and who consequently may have benefited from diagnosis and treatment of their depression symptoms (Table 4). Receipt of any treatment (antidepressant medication, counselling or psychiatric referral) was more likely in participants with higher CESD-10 scores in every model. Antidepressant medication at therapeutic doses at baseline was more likely in participants with more education, higher income, unemployed, or in clinics with a pharmacist, and was less likely in males and Xhosa speakers, independently of their baseline CESD-10 score. In the case of education, there appeared to be a dose–response relationship, indicated by a steady increase in treatment access with more years of education. Receipt of therapeutic doses of antidepressant drug at follow-up was more likely in women, participants with higher income or in clinics with a pharmacist, drugs supplied off-site, daily doctor support, lower patient to nurse ratios, or peri-urban or rural location, independently of baseline CESD-10 score and antidepressant medication.

**Table 4:** Baseline health, socioeconomic and clinic characteristics independently associated with mental health treatments at baseline and at follow-up^a^, in patients with CESD-10 score of ten or more: logistic regression models

Outcome	Therapeutic dose of antidepressant drug at baseline				Therapeutic dose of antidepressant drug at follow-up^a^				Psychiatric referral between baseline and follow-up				Counselling between baseline and follow-up			
Explanatory variable	OR^b^	95 % CI^b^		*P*	OR	95 % CI		*P*	OR	95 % CI		*P*	OR	95 % CI		*P*
Patient characteristics																
Age (per year)									0.97	0.95	0.98	<0.001	0.97	0.96	0.98	<0.001
Men vs. women	0.31	0.19	0.49	<0.001	0.34	0.22	0.53	<0.001	0.67	0.43	1.03	0.069				
Hypertension													0.75	0.60	0.95	0.016
Diabetes	0.72	0.49	1.06	0.094	0.71	0.48	1.04	0.079								
Highest education				0.002^c^								0.006^c^				0.022^c^
•None (reference)	1.00								1.00				1.00			
•Primary	1.36	0.75	2.47	0.314					1.11	0.51	2.38	0.797	0.85	0.51	1.41	0.529
•Secondary	1.98	1.10	3.55	0.022					1.37	0.62	2.99	0.435	0.81	0.49	1.33	0.401
•Tertiary	2.06	0.91	4.66	0.082					4.00	1.57	10.15	0.004	1.99	1.08	3.68	0.028
Language				<0.001^c^								0.003^c^				
•Afrikaans (reference)	1.00								1.00							
•Xhosa	0.23	0.15	0.35	<0.001					0.16	0.05	0.45	0.001				
•English	0.85	0.54	1.35	0.488					1.10	0.64	1.90	0.732				
Income (per 1000 rand per month)	1.31	1.18	1.44	<0.001	1.21	1.03	1.43	0.023	1.19	1.05	1.34	0.005				
Unemployed	2.01	1.32	3.09	0.001												
Welfare grant									1.35	0.96	1.90	0.082	1.36	1.13	1.64	0.001
Baseline CESD-10 score (per unit)	1.15	1.12	1.19	<0.001	1.06	1.00	1.11	0.033	1.11	1.06	1.15	<0.001	1.06	1.03	1.10	<0.001
Therapeutic dose of antidepressant at baseline^a^	NA^b^				136.90	77.25	242.61	<0.001	NA				NA			
Clinic characteristics																
Pharmacist in clinic	1.86	1.25	2.78	0.002	1.58	0.99	2.51	0.053								
Drug supply available away from clinic					1.67	1.11	2.50	0.013					1.59	0.95	2.66	0.078
Psychiatric nurse at clinic													0.32	0.13	0.80	0.015
Doctor at clinic every day					1.48	0.95	2.30	0.084								
Clinic location								0.038^c^								
•Urban (reference)					1.00											
•Peri-urban					1.17	0.66	2.08	0.599								
•Rural					2.60	1.18	5.76	0.018								
Clinic patients per year/10,000													1.04	0.99	1.10	0.081
Clinic patients per nurse per year/1000					0.93	0.88	0.99	0.022								
Intervention vs. control clinic					1.39	0.97	1.98	0.073	0.57	0.35	0.91	0.020	0.54	0.33	0.88	0.013

^a^ Change modelled with analysis of covariance, that is, with baseline value as covariate

^b^ *OR* odds ratio, *CI* confidence interval, *NA* not applicable

^c^ Wald test for all categories of variable

Psychiatric referral between baseline and follow-up was more likely in participants with tertiary education or higher income and was less likely in participants who were older, male, Xhosa-speaking or in intervention clinics.

Counselling between baseline and follow-up was more likely in participants with more education or receiving welfare grants, and in clinics that supplied drugs away from the clinics, and was less likely in participants who were older or had hypertension, and in intervention clinics or clinics with a psychiatric nurse.

## Discussion

This study shows that depression symptoms in adults attending primary care clinics in two districts of South Africa, most of whom had common chronic conditions, were strongly and independently associated with several indicators of disadvantaged socioeconomic position. Depression symptoms, as indicated by higher CESD-10 scores at baseline, were independently associated with being less educated and having lower income. CESD-10 scores at follow-up had increased since baseline in participants who were less educated or receiving welfare grants. Level of education was however not associated with baseline CESD-10 score in the crude analysis, being confounded by the other socioeconomic indicators. This is consistent with findings from several other LMICs, where education was less frequently associated with common mental disorders in bivariate analyses than in multivariate analyses [11].

Previous studies, the majority of which have been community based, have similarly demonstrated associations between common mental disorders and socioeconomic factors, including less education [11, 13, 33, 34], low socio-economic status [11] and low income [13, 34].

Our study population comprised patients already using primary care facilities, and therefore relatively easy to reach for diagnosis and treatment of depression. It showed that, at baseline, participants were less likely to have received treatment with antidepressants if they were socially disadvantaged, in particular if they had lower income or less education. However, participants were more likely to receive treatment if they were unemployed. This may be because it is easier for unemployed participants to attend clinics for treatment. In contrast, the SASH study found no significant associations between receiving treatment for mental disorders and income or level of education [35]. At follow-up, clinic characteristics were more important than socio-economic factors in predicting depression treatment, with participants less likely to have received antidepressant medication if they attended less resourced clinics, without a pharmacist or off-site drug delivery, or if they had lower income. Primary care clinics should be adequately staffed and have pharmacists on site but also enable patients to collect their repeated medicines at more convenient locations. Strategies to deal with the shortage of doctors and nurses in the South African public sector, especially in rural areas, have included community service for doctors, monetary incentives, introducing a cadre of mid-level workers such as pharmacists, contracting non-professional health workers to take on various responsibilities such as counselling and adherence support, and introducing innovative clinical guidelines to enable nurses to manage patients who would otherwise be seen by doctors [36]. Nevertheless, our results reflect the effects of variation in patient:staff ratios within a resource-constrained system, and suggest the need to equalise workloads between clinics, with existing resources. Our finding that patients in clinics with a psychiatric nurse were less likely to receive counselling at follow-up is counter-intuitive. It may be that psychiatric nurses are managing patients with more severe psychiatric disease, that is, psychoses mostly treated with drugs, and do not have the time or skills to provide counselling for depression.

Patients who were more depressed at baseline were more likely to receive antidepressant medication subsequently. Causal inference about the cross-sectional association between depression symptoms at baseline and treatment at baseline is not as clear, but it is more plausible that depression led to treatment rather than that treatment led to depression. Women were more likely than men to have higher CESD-10 scores and to receive treatment with antidepressants at baseline and follow-up. These findings are consistent with work from HIV cohorts in Southern Africa which have shown that proportionally more women than men are on antiretroviral therapy [37], and highlights the need to identify barriers to men accessing healthcare [38, 39]. The role of gender in the causation, experience, reporting and care of depression is however an enormous subject which was beyond the scope of this study.

Depression could potentially have affected participants’ physical health through biological mechanisms, or through their health care use, treatment adherence or interpretation of physical symptoms. However, we found that depression symptoms at baseline were not associated with changes in blood pressure control, glycaemic control, respiratory symptom score or ten year risk of death from cardiovascular disease. This differs from studies which have shown a positive association between depression and poor glycaemic control in diabetic patients [14].

Our findings suggest that the association between depression symptoms and socio-economic position is bidirectional. That is, in addition to disadvantaged social position predicting worse depression symptoms at follow-up, participants who had depression symptoms at baseline were more socially disadvantaged at follow-up, showing 25 % higher odds of being unemployed. The bidirectional link between depression symptoms and social disadvantage therefore supports both the social causation and social selection theories. Our findings suggest that, in this study setting, socioeconomic disadvantage is both a cause and a consequence of depression, and may also be a barrier to treatment, with participants less likely to receive treatment if they had a lower income (baseline and follow-up) or less education (baseline).

The study had a number of strengths. The sample size was large, high rates of follow-up were achieved, and a wide range of socio-economic variables were investigated. A key strength of the study was the longitudinal design, which allowed potential causal relationships to be identified, demonstrating that the relationship between socioeconomic position and depression worked in both directions.

There were a number of limitations to the study. The CESD-10 questionnaire was used to identify participants with depression symptoms, but not to confirm the clinical diagnosis of depression. It was originally derived and validated on an older adult population [22] but has subsequently been validated in a younger population on antiretroviral therapy [25]. Participants were only enrolled into the study if they had hypertension, diabetes, chronic respiratory disease or depression symptoms, so the results may not be generalisable to primary care attenders without these conditions. Thirty-four percent of participants in the depression group did not answer the question at baseline on whether they had received counselling in the past year. This was due to an error in the electronic questionnaire that resulted in this question being skipped during several weeks of fieldwork before it was detected and corrected. Socioeconomic factors that could influence depression symptoms that were not measured include food insecurity, poor housing, lack of social support, and disability.

Further research is needed to investigate the relative contributions of both social causation and social selection/drift mechanisms to the well documented association between socio-economic disadvantage and depression in LMICs; to identify what specific intervention strategies are needed to reach vulnerable low socioeconomic populations living with depression; and to evaluate the effectiveness of interventions that are designed to target each of the above mechanisms. Feasible examples might include brief psychological interventions with financial risk protection as part of universal health coverage.

## Conclusion

This study provides new evidence from South Africa in support of the bidirectional relationship between poverty and depression. Mental health interventions have been shown to be associated with improved economic outcomes in LMICs [16]. This study reinforces arguments for the expansion of mental health services and improving the prevention, detection and treatment of depression in primary health care settings in South Africa and other LMICs, for clinical and economic reasons. While there is currently an emphasis on integrating communicable and non-communicable chronic disease care in South Africa, we must not lose sight of the importance of ensuring better management and access to mental health care.

## References

[1] Ferrari AJ, Charlson FJ, Norman RE, Patten SB, Freedman G, Murray CJL (2013). Burden of depressive disorders by country, sex, age, and year: findings from the global burden of disease study 2010. PLoS Med.

[2] Vos T, Flaxman AD, Naghavi M, Lozano R, Michaud C, Ezzati M (2012). Years lived with disability (YLDs) for 1160 sequelae of 289 diseases and injuries 1990–2010: a systematic analysis for the Global Burden of Disease Study 2010. Lancet.

[3] Tomlinson M, Grimsrud AT, Stein DJ, Williams DR, Myer L (2009). The Epidemiology of Major Depression in South Africa: Results from the South African Stress and Health Study. S Afr Med J.

[4] Health Systems Trust. South African Health Review 2010. Durban: Health Systems Trust, 2010. http://www.hst.org.za/publications/south-african-health-review-2010. Accessed 14 August 2014.

[5] Williams DR, Herman A, Stein DJ, Heeringa SG, Jackson PB, Moomal H (2008). Twelve-month mental disorders in South Africa: prevalence, service use and demographic correlates in the population-based South African Stress and Health Study. Psychol Med.

[6] World Health Organisation. Mental Health Gap Action Programme (mhGAP) 2010. http://www.who.int/mental_health/mhgap/en/. Accessed 11 November 2014.

[7] Tregenna F, Tsela M (2012). Inequality in South Africa: The distribution of income, expenditure and earnings. Dev South Afr.

[8] Benatar SR (2013). The challenges of health disparities in South Africa. S Afr Med J.

[9] Messias E, Eaton WW, Grooms AN (2011). Economic Grand Rounds: Income Inequality and Depression Prevalence Across the United States: An Ecological Study. Psychiatr Serv.

[10] Muramatsu N (2003). County-level income inequality and depression among older Americans. Health Serv Res.

[11] Lund C, Breen A, Flisher AJ, Kakuma R, Corrigall J, Joska JA (2010). Poverty and common mental disorders in low and middle income countries: A systematic review. Soc Sci Med.

[12] Ataguba JE, Akazili J, McIntyre D (2011). Socioeconomic-related health inequality in South Africa: evidence from General Household Surveys. Int J Equity Health.

[13] Patel V, Kleinman A (2003). Poverty and common mental disorders in developing countries. Bull World Health Organ.

[14] Prince M, Patel V, Saxena S, Maj M, Maselko J, Phillips MR (2007). No health without mental health. Lancet.

[15] Parreira VF, Kirkwood RN, Towns M, Aganon I, Barrett L, Darling C (2015). Is There an Association between Symptoms of Anxiety and Depression and Quality of Life in Patients with Chronic Obstructive Pulmonary Disease?. Can Respir J.

[16] Lund C, De Silva M, Plagerson S, Cooper S, Chisholm D, Das J (2011). Poverty and mental disorders: breaking the cycle in low-income and middle-income countries. Lancet.

[17] Dohrenwend BP, Levav I, Shrout PE, Schwartz S, Naveh G, Link BG (1992). Socioeconomic status and psychiatric disorders: the causation-selection issue. Science.

[18] Health Systems Trust. Primary Health Care 101. 2013. http://www.hst.org.za/publications/primary-health-healthcare-101. Accessed 22 November 2015.

[19] Folb N, Timmerman V, Levitt NS, Steyn K, Bachmann MO, Lund C (2015). Multimorbidity, control and treatment of non-communicable diseases among primary healthcare attenders in the Western Cape, South Africa. S Afr Med J.

[20] Statistics South Africa. Census 2011 Municipal factsheet. http://www.statssa.gov.za/census/census_2011/census_products/Census_2011_Municipal_fact_sheet.pdf. Accessed 22 November 2015.

[21] Eden District Municipality. Eden District Municipality Integrated Development Plan (IDP) Annual Review for 2010/11. Eden District Municipality, 2010. http://mfma.treasury.gov.za/Documents/01.%20Integrated%20Development%20Plans/2010-11/03.%20District%20Municipalities/DC4%20Eden/DC4%20Eden%20-%20IDP%20-%201011.pdf. Accessed 21 August 2014.

[22] Andresen EM, Malmgren JA, Carter WB, Patrick DL (1994). Screening for depression in well older adults: evaluation of a short form of the CES-D (Center for Epidemiologic Studies Depression Scale). Am J Prev Med.

[23] Radloff LS (1977). The CES-D Scale: A self-report depression scale for research in the general population. Appl Psychol Meas.

[24] Miller WC, Anton HA, Townson AF (2008). Measurement properties of the CESD scale among individuals with spinal cord injury. Spinal Cord.

[25] Zhang W, O’Brien N, Forrest JI, Salters KA, Patterson TL, Montaner JSG (2012). Validating a shortened depression scale (10 item CES-D) among HIV-positive people in British Columbia, Canada. PLoS One.

[26] Myer L, Smit J, Roux LL, Parker S, Stein DJ, Seedat S (2008). Common mental disorders among HIV-infected individuals in South Africa: prevalence, predictors, and validation of brief psychiatric rating scales. AIDS Patient Care STDs.

[27] Rossiter D, editor. South African Medicines Formulary (SAMF). Health and Medical Publishing Group of the South African Medical Association. Cape Town, South Africa. 11th edition; 2014.

[28] Gaziano TA, Young CR, Fitzmaurice G, Atwood S, Gaziano JM (2008). Laboratory-based versus non-laboratory-based method for assessment of cardiovascular disease risk: the NHANES I Follow-up Study cohort. Lancet.

[29] Jones PW, Quirk FH, Baveystock CM (1991). The St George’s Respiratory Questionnaire. Respir Med.

[30] StataCorp (2011). Stata Statistical Software: Release 12.

[31] Barnett AG, van der Pols JC, Dobson AJ (2005). Regression to the mean: what it is and how to deal with it. Int J Epidemiol.

[32] OANDA currency converter. http://www.oanda.com/currency/historical-rates/. Accessed 24 December 2013.

[33] Araya R, Lewis G, Rojas G, Fritsch R (2003). Education and income: which is more important for mental health?. J Epidemiol Community Health.

[34] Patel V, Araya R, de Lima M, Ludermir A, Todd C (1999). Women, poverty and common mental disorders in four restructuring societies. Soc Sci Med.

[35] Seedat S, Stein DJ, Herman A, Kessler R, Sonnega J, Heeringa S (2008). Twelve-month treatment of psychiatric disorders in the South African Stress and Health Study (World Mental Health Survey Initiative). Soc Psychiatry Psychiatr Epidemiol.

[36] Daviaud E, Chopra M (2008). How Much Is Not Enough? Human Resources Requirements for Primary Health Care: A Case Study from South Africa. Bull World Health Organ.

[37] Muula AS, Ngulube TJ, Siziya S, Makupe CM, Umar E, Prozesky HW (2007). Gender distribution of adult patients on highly active antiretroviral therapy (HAART) in Southern Africa: a systematic review. BMC Public Health.

[38] Galdas PM, Cheater F, Marshall P (2005). Men and Health Help-Seeking Behaviour: Literature Review. J Adv Nurs.

[39] Möller-Leimkühler AM (2002). Barriers to Help-Seeking by Men: A Review of Sociocultural and Clinical Literature with Particular Reference to Depression. J Affect Disord.

